# Childbirth-related post-traumatic stress disorder symptoms and mother–infant neurophysiological and behavioral co-regulation during dyadic interaction: study protocol

**DOI:** 10.1186/s40359-023-01070-0

**Published:** 2023-02-09

**Authors:** Tiago Miguel Pinto, Inês Jongenelen, Diogo Lamela, Rita Pasion, Ana Morais, Raquel Costa

**Affiliations:** 1 Digital Human-Environment Interaction Labs (HEI-Lab), Lusófona University, R. de Augusto Rosa 24, 4000-098 Porto, Portugal; 2 EPIUnit, Instituto de Saúde Pública, Universidade do Porto, Rua das Taipas, 135, 4050-600 Porto, Portugal; 3 Laboratório para a Investigação Integrativa e Translacional em Saúde Populacional (ITR), Porto, Portugal

**Keywords:** Childbirth, Postpartum, Post-traumatic stress disorder, Mother–infant interaction, Mother–infant co-regulation, Neurophysiological co-regulation, Behavioral co-regulation, Previous trauma, Anxiety, Depression

## Abstract

**Background:**

Mother’s childbirth-related posttraumatic stress disorder (PTSD) symptoms have a negative impact on mother and infant’s behaviors during dyadic interactions which may increase mother–infant neurophysiological and behavioral co-regulation difficulties, leading to dysregulated mother–infant interactions. This study was specifically designed to analyze: (1) the sociodemographic and obstetric factors associated with mother’s childbirth-related PTSD symptoms; (2) mother–infant neurophysiological functioning and behavioral co-regulation during dyadic interaction; (3) the impact of mother’s childbirth-related PTSD symptoms on neurophysiological and behavioral mother–infant co-regulation during dyadic interaction; (4) the moderator role of previous trauma on the impact of mother’s childbirth-related PTSD symptoms on neurophysiological and behavioral mother–infant co-regulation during dyadic interaction; and (5) the moderator role of comorbid symptoms of anxiety and depression on the impact of mother’s childbirth-related PTSD symptoms on neurophysiological and behavioral mother–infant co-regulation during dyadic interaction.

**Methods:**

At least 250 mothers will be contacted in order to account for refusals and dropouts and guarantee at least 100 participating mother–infant dyads with all the assessment waves completed. The study has a longitudinal design with three assessment waves: (1) 1–3 days postpartum, (2) 8 weeks postpartum, and (3) 22 weeks postpartum. Between 1 and 3 days postpartum, mothers will report on-site on their sociodemographic and obstetric characteristics. At 8 weeks postpartum, mothers will complete online self-reported measures of birth trauma, previous trauma, childbirth-related PTSD, anxiety, and depressive symptoms. At 22 weeks postpartum, mothers will complete online self-reported measures of childbirth-related PTSD, anxiety, and depressive symptoms. Mothers and infants will then be home-visited to observe and record their neurophysiological, neuroimaging and behavioral data during dyadic interactions using the Still-face Paradigm. Activation patterns in the prefrontal cortices of mother and infant will be recorded simultaneously using hyperscanning acquisition devices. Unadjusted and adjusted multilevel linear regression models will be performed to analyze objectives 1 to 3. Moderation models will be performed to analyze objectives 4 and 5.

**Discussion:**

Data from this study will inform psychological interventions targeting mother–infant interaction, co-regulation, and infant development. Moreover, these results can contribute to designing effective screenings to identify mothers at risk of perinatal mental health problems and those who may need specialized perinatal mental health care.

## Background

Childbirth can be a traumatic event as it can threaten the life of the mother and/or her infant and lead to childbirth-related posttraumatic stress disorder (PTSD) [1]. This is an important public health issue since the estimated prevalence can go up to 4% in community samples and 16% in risk samples [1, 2]. In 2020, 84 426 gave birth in Portugal [3], which means that at least about 3 377 women may have experienced childbirth-related PTSD.

According to the Developmental Psychopathology framework, the environment plays a major role in infant development [4]. Namely, mother’s childbirth-related PTSD symptoms have an adverse impact on infant self-regulation development [5]. Infants of mothers with more severe postpartum PTSD symptoms seem to experience increased challenges in the development of self-regulatory mechanisms, a major developmental task that allows the infant to adequately respond to environmental demands [6, 7]. They have lower neurophysiological and behavioral regulation, namely lower salivary cortisol levels, more sleeping difficulties, lower ability to recover from distress, and more behavior dysregulation symptoms [8–10]. Importantly, the development of infant neurophysiological and behavioral regulation abilities is mainly supported by caregiver-infant co-regulation during interaction [11].

Caregiver-infant co-regulation is defined as the process by which caregivers dynamically change their behavior to anticipate and adequately respond to the ongoing behavior of their infant, allowing positive caregiver-infant interaction and communication [12]. Sensitivity—the ability to identify and adequately respond to the infant signals both in non-distress and distress situations—, is an important parental characteristic to promote the successful development of infant self-regulation [13]. Lower maternal sensitivity during mother–infant interaction may be the path that explains the negative impact of childbirth-related PTSD symptoms on the development of infant neurophysiological and behavioral regulation abilities, as it can lead to lower mother–infant co-regulation.

Mother’s childbirth-related PTSD symptoms have indeed a negative impact on the mother’s and infant’s behaviors during dyadic interactions [6, 14, 15]. Mothers with more PTSD symptoms have lower sensitivity and higher control while interacting with their infants, compared to mothers with fewer PTSD symptoms [14]. The Still Face Paradigm (SFP), a procedure to assess caregiver sensitivity in response to infant signals both in non-distress and distress-induced situations, provides important insights into mother’s and infant’s behaviors during dyadic interactions [16]. Mothers with more postpartum PTSD symptoms tend to not look directly, to repeatedly touch their infant, and to negatively describe their infant’s status, compared to mothers with fewer PTSD symptoms. Infants of mothers with more PTSD symptoms display (1) more avoidance and disorganized behavior (distress, uncontrollable, inconsolable crying) and less interest in the toys presented in the SFP play episode (non-distress situation), (2) more eye contact avoidance and more physical avoidance behaviors towards their mothers in the SFP still episode (distress-induced situation), and (3) lower recovery from distress, namely harder crying in the SFP reunion episode [6, 15].

An unexplored hypothesis is that mother’s childbirth-related PTSD symptoms may have a negative impact on the quality of the dyadic interaction by disrupting mother–infant brain synchrony and co-regulation [17]. Synchrony in biological rhythms and social signals is an evolutionary adaptive mechanism of bond formation. It arises through continuous mutual adaptations and is first experienced with the caregiver, shaping the developing brain during early sensitive periods [18, 19]. While there is some evidence about mother–infant behavioral synchrony, less is known about the neurophysiological mechanisms supporting brain-to-brain coordination, and mother–infant neurophysiological co-regulation.

Although underexplored, mother’s childbirth-related PTSD symptoms may increase dysregulated mother–infant interactions, due to lower mother–infant co-regulation during interactions, both at neurophysiological and behavioral levels. Recent studies show that parent and infant's brain activities synchronize in anterior and posterior prefrontal regions during cooperation, and these regions correlate with the mother’s stress and mediate the association between parents’ and infants’ emotional regulation [17, 20, 21]. When interacting with their infants, mothers with more PTSD symptoms may exhibit less dyadic prefrontal cortical synchrony, having more difficulties regulating their neurophysiological and behavioral states to adequately respond to infant signals, resulting in lower sensitivity. This may disconnect infants from their mothers during the interaction, reducing prefrontal activation and increasing avoidant behaviors, as they may fail to recruit the necessary prefrontal areas for top-down processes of regulation.

Addressing this innovative hypothesis, this study—CORE-PTSD—was specifically designed to analyze the impact of mother’s childbirth-related PTSD symptoms on mother–infant neurophysiological and behavioral co-regulation during dyadic interaction. Both mother–infant neurophysiological and behavioral co-regulation will be assessed during the SFP, which is a major innovation of the CORE-PTSD. Previous trauma, postpartum anxiety and depressive symptoms seem to be associated with more postpartum PTSD symptoms in mothers, lower quality in mother–infant interaction, and lower regulation in infants [22]. Considering this evidence and the high comorbidity among perinatal mental health disorders [23], previous trauma and postpartum anxiety and depressive symptoms will be included in the models as moderators of the impact of mother’s childbirth-related PTSD symptoms on mother–infant neurophysiological and behavioral co-regulation during dyadic interaction, which is also a major innovation of CORE-PTSD.

**Fig. 1:**
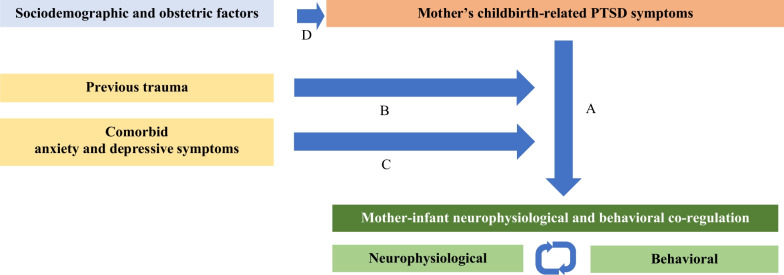
Model to analyze the impact of mother’s childbirth-related PTSD symptoms on mother–infant neurophysiological and behavioral co-regulation during dyadic interaction. Path B and C represent the moderation role of the previous trauma and comorbid anxiety and depressive symptoms on the Path A. Path D represents the potential co-variates adjustments in Paths A, B, and C

As such, the main aim of the CORE-PTSD is to analyze the impact of mother’s childbirth-related PTSD symptoms on mother–infant neurophysiological and behavioral co-regulation during dyadic interaction. Specific aims are to analyze: (1) the sociodemographic and obstetric factors associated with mother’s childbirth-related PTSD symptoms; (2) mother–infant neurophysiological functioning and behavioral co-regulation during dyadic interaction; (3) the impact of mother’s childbirth-related PTSD symptoms on neurophysiological and behavioral mother–infant co-regulation during dyadic interaction; (4) the moderator role of previous trauma on the impact of mother’s childbirth-related PTSD symptoms on neurophysiological and behavioral mother–infant co-regulation during dyadic interaction; and (5) the moderator role of comorbid symptoms of anxiety and depression on the impact of mother’s childbirth-related PTSD symptoms on neurophysiological and behavioral mother–infant co-regulation during dyadic interaction. The graphic representation of the CORE-PTSD model is presented in Fig. 1.

## Methods/design

### Participants

We expect to contact at least 250 mothers in order to account for refusals and dropouts and guarantee at least 100 participating mother–infant dyads with all the assessment waves completed. Mothers will be eligible if they are 18 years of age or older, with a single gestation of ≥ 37 weeks, and infants with birth weight ≥ 2500 g and without congenital abnormalities. À priori power calculations performed following recommendations [24] revealed that the sample size is adequate to conduct linear regression models (*f*^2^ = 0.25, *p* < 0.05, *N* = 85, number of predictors = 5; power = 0.95) in order to address the proposed aims.

### Procedures

The study was approved by the Lusófona University Ethical Committee and by the ethical committees of the Health Facilities in which the recruitment will be conducted. A researcher will contact eligible mothers after childbirth before hospital discharge and will explain the objectives and procedures of the study. Mothers will be informed about the study purposes and procedures, will be invited to participate, and those willing to participate will sign an informed consent. This study has a longitudinal design with three assessment waves: (1) 1–3 days postpartum, (2) 8 weeks postpartum, and (3) 22 weeks postpartum. Between 1 and 3 days postpartum, mothers will report on-site on their sociodemographic and obstetric characteristics. At 8 weeks postpartum, mothers will complete online self-reported measures of birth trauma, previous trauma, childbirth-related PTSD symptoms, and anxiety and depressive symptoms. At 22 weeks postpartum, mothers will complete online self-reported measures of childbirth-related PTSD symptoms, and anxiety and depressive symptoms. Mothers and infants will then be home-visited to observe and record their behavioral co-regulation during the SFP [16]. Neurophysiological and neuroimaging data will be collected at this stage, at their home (naturalist setting), since synchrony between mothers and their infants has been observed as early as 3 months postpartum during face-to-face interactions [17]. Mothers and infants will be observed and recorded during a 5-min face-to-face interaction, that will be coded with the Global Rating Scales of Mother–infant Interaçtion (GRS) [25, 26] to assess mother and infant’s baseline neurophysiological and behavioral regulation. Afterward, the SFP will be conducted. In the first episode, the mother will be asked to play without toys with the infant. In the second episode, the mother will be asked to show a still face or lack of response to the infant for 2 min. Finally, mother–infant interaction is repaired in the 3rd episode, by asking the mother to return to normal and return to playing and talking to the infant.

During SFP, activation patterns in the prefrontal cortices of the mother and infant will be recorded simultaneously using hyperscanning acquisition devices. Functional near-infrared Spectroscopy (fNIR) will be collected from PLUX Wireless Biosignals (Biosignalplux, Hybrid-8). fNIR sensors of this device (sampling rate of 500 Hz) have a resolution of 24-bit and a dual-LED design—1 red (wavelength: 660 nm) and 1 infrared LED (wavelength: 950 nm). Emitters and detectors (sensitivity: 400–110 nm max@920 nm) are separated by 2 cm. Galvanic skin response will be measured as a metric of emotional activation/distress in SFP using a wireless device (James II, Mindprober, PT, sampling rate: 500 Hz). Both apparatuses are well-validated and have small/non-invasive sensors. They require minimal setup time and capture high-quality biometric signals in naturalistic settings (i.e., artifact-resistant, and high signal-to-noise ratio). They will be set up following general procedures to minimize any interference with the interaction (e.g., placing a forehead hand and a hand band) [27, 28]. Conditions of the SFP (play episode, still episode, and reunion episode) will be the triggers for both devices. The study’s design, assessment waves, variables, and measures are presented in Table 1.

**Table 1:** Study design, assessments, variables, and measures

Study variables	Postpartum		
	1–3 days	8 weeks	22 weeks
Sociodemographic, obstetric, and clinical data	Sociodemographic and obstetric information^a^	Birth trauma^b^	
		Previous trauma^c^	
Mother’s perinatal mental health problems		Childbirth-related PTSD symptoms^d^	Childbirth-related PTSD symptoms^d^
		Depressive symptoms^e^	Depressive symptoms^e^
		Anxiety symptoms^f^	Anxiety symptoms^f^
Behavioral mother–infant co-regulation			Mother–infant interaction^g^
			Still-Face Paradigm^h^
Neurophysiological mother–infant co-regulation			Skin Conductance^i^
			Neuroimaging on mother–infant brain synchrony^j^

^a^Questionnaire on sociodemographic and obstetric data; ^b^Childbirth-related trauma item; ^c^Diagnostic Scale of Post-traumatic Stress Disorder; ^d^City Birth Trauma Scale; ^e^Edinburgh Postnatal Depression Scale; ^f^State-Trait Anxiety Inventory; ^g^Global Rating Scales of Mother–infant Interaçtion; ^h^Still-face paradigm; ^i^MindProber’s biometric sensors; ^j^Plux fNIR

### Measures

#### Sociodemographic and obstetric information

Demographic and obstetric data will be collected using a self-reported questionnaire and includes age, educational level, marital status, professional status, country of birth, country of residence, parity, gestational age at birth, type of birth, birth weight, infant sex, and maternal/neonatal complications.

#### Birth trauma

Childbirth-related trauma will be assessed using a single item scored on a 10-point Likert-type scale related to childbirth: “Not traumatic at all” (0) to “Extremely traumatic” (10).

#### Childbirth-related PTSD

The City Birth Trauma Scale (City BiTS) will be used [29, 30]. The City BiTS comprises 29 questions that map onto the Diagnostic and Statistical Manual of Mental Disorders-5 (DSM-5) diagnostic criteria. Symptoms are rated for frequency over the last week and scored on a scale ranging from 0 (‘not at all’) to 3 (‘5 or more times’). Higher scores indicate greater symptoms of PTSD. Diagnostic criterion A items are scored on a yes/no scale. Distress, disability, and potential physical causes are rated as yes/no/maybe.

#### Previous trauma

The Diagnostic Scale of PTSD will be used [31]. Previous trauma and involuntary pregnancy interruption and stillbirth will also be assessed.

#### Postpartum depression symptoms

The Edinburgh Postnatal Depression Scale (EPDS) will be used [32, 33]. The EPDS is a 10-item self-report scale, scored on a 4-point Likert-type scale (0–3), that was designed to assess depressive symptoms within the previous 7 days and to screen for postpartum depression. Higher scores indicate greater symptom severity.

#### Postpartum anxiety symptoms

The State-Trait Anxiety Inventory (STAI-S/T) will be used [33, 34]. The STAI-S/T comprises two 20-item scales, scored on a 4-point Likert-type scale, designed to assess state and trait anxiety symptoms. Higher scores indicate greater symptom severity.

#### Mother–infant co-regulation

##### Behavioral

Mother–Infant Interaction. The GRS will be used to assess the quality of mother–infant interaction [25, 26]. It consists of a 5-min face-to-face, mother–infant video-recorded interaction without the use of toys, that is scored according to the GRS manual instructions.

Still-face paradigm. SFP will be used to assess mother–infant co-regulation during dyadic interaction [16]. It comprises 3 episodes: (1) a face-to-face play episode (5 min); (2) a still-face episode (2 min), during which the caregiver does not respond to the infant while holding a neutral expression; and (3) a reunion episode, during which the caregiver repairs the interaction with the infant, often distressed.

#### Neurophysiological

Galvanic Skin Conductance. MindProber’s biometric sensors collect the galvanic skin response, a metric of emotional activation. A meta-analysis has shown indeed that skin conductance levels are the single most reliable indicator of emotional arousal and allow to measure short-latency responses [35]. We will compute simultaneously to mothers and infants both tonic (slow changes, seconds to minutes) and phasic (rapid chances, seconds) variations across SPF conditions (play phase, still phase, and reunion phase).

Neuroimaging. Plux fNIR solutions provide a hyper scanning mode to measure between-person coordination brain activity dynamics, as indicated by the alignment of two independent signals across time (i.e., SFP task conditions—play phase, still phase, and reunion phase). The prefrontal cortex will be targeted as it is associated with active top-down regulation of emotional responses. The sensors will be placed in both right and left anterior and posterior regions (10–20 system: F3, F4, Fp1, Fp2) for both mother and infant. Mean concentration changes in oxygenated hemoglobin (HbO) and deoxygenated hemoglobin (HbR) levels in the cortical surface for each SPF condition and participant will be calculated. Higher HbO (and lower HbR) reflects increased cerebral blood flow to meet metabolic requirements (i.e., reflects higher brain activation). At the dyadic level, wavelet transformation coherences of the HbO and HbR between mother and infant will be computed for each condition [36, 37]. It is an indicator of neural synchrony by measuring the cross-correlation of two-time series based on the continuous wavelet transform at the given frequency and time.

### Data analysis

The aims of the CORE-PTSD will be achieved using descriptive and multivariate analysis. Unadjusted and adjusted multilevel linear regression models will be performed to analyze specific aims 1–3. Moderation models will be performed to analyze specific aims 4 and 5. The statistical assumptions to conduct the multilevel linear regression models will be analyzed. The statistical analysis will be performed using the IBM SPSS 28.0

## Discussion

Findings of the CORE-PTSD will provide evidence on the impact of mother’s childbirth-related PTSD symptoms on mother–infant neurophysiological and behavioral co-regulation during dyadic interaction, which can inform psychological interventions targeting mother–infant interaction and co-regulation and infant development, namely, to promote infant self-regulation development or to prevent or treat self-regulation problems.

CORE-PTSD findings will provide evidence on the sociodemographic and obstetric factors associated with the mother’s perinatal mental health problems, namely the mother’s childbirth-related PTSD symptoms, and postpartum anxiety and depressive symptoms. It provides data according to individual, family, cultural and socio-economic factors. These results contribute to developing more effective screenings to identify early during pregnancy mothers at risk of perinatal mental health problems and those who may need specialized perinatal mental health care. This information allows timely referral and appropriate allocation of resources to face local unmet perinatal mental health care needs attending to families’ demands and contributing to improved and inclusive public perinatal mental healthcare preparedness. This aligns with the 2030 Sustainable Development Agenda from the United Nations; specifically, goal 3.4—reduce by one-third premature mortality from non-communicable diseases through prevention and treatment and promote mental health and wellbeing.

Neurophysiological and behavioral data collected during dyadic interactions can further inform early interventions on mother–infant interaction and co-regulation, which is once again aligned with goal 3.4 to reducing premature mortality from non-communicable disease in the infant. For instance, emotional dysregulation is a transdiagnostic feature of mental health problems at all ages [38], that are, in turn, associated with death by suicide [39]. Moreover, emotional dysregulation is associated with externalizing problems across the lifespan, namely substance abuse [40] which also associated with death by suicide [39]. Thus, findings specifically inform early intervention aiming to prevent emotion regulation problems, which also aligns with goal 3.5 from the 2030 Sustainable Development Agenda of United Nations—strengthen the prevention and treatment of substance abuse, including narcotic drug abuse and harmful use of alcohol.

The CORE-PTSD results will be communicated and disseminated within the non-scientific and scientific communities. Within the non-scientific community, periodical newsletters are developed and posted in relevant social networks, namely key findings, fact sheets, or research briefs. Within the scientific community, the CORE-PTSD results will be communicated and disseminated in national and international research events, international papers, and the presentation of oral and poster communications in national and international congresses, and through master and Ph.D. theses.

## Data Availability

Available upon request to the corresponding author.

## Abbreviations

PTSD: Posttraumatic stress disorder
SFP: Still Face Paradigm
GRS: Global Rating Scales of Mother–infant Interaçtion
fNIR: Functional near-infrared spectroscopy
City BiTS: City Birth Trauma Scale
DSM-5: Diagnostic and Statistical Manual of Mental Disorders-5
EPDS: Edinburgh Postnatal Depression Scale
STAI-S/T: State-Trait Anxiety Inventory
HbO: Oxygenated hemoglobin
HbR: Deoxygenated hemoglobin
